# Long non-coding RNA SPRY4-IT1 promotes gallbladder carcinoma progression

**DOI:** 10.18632/oncotarget.13621

**Published:** 2016-11-25

**Authors:** Liang Yang, Xi Cheng, Naijian Ge, Weixing Guo, Feiling Feng, Fuying Wan

**Affiliations:** ^1^ Radiation Center, East Hepatobiliary Surgery Hospital, Second Military Medical University, Shanghai, 201805, China; ^2^ Mini-Invasive Intervention Center, Eastern Hepatobiliary Surgery Hospital, Second Military Medical University, Shanghai, 200438, China; ^3^ Hepatic Surgical Department VI, Eastern Hepatobiliary Surgery Hospital, The Second Military Medical University, Shanghai, 200438, China; ^4^ Department of Biliary Tract, Eastern Hepatobiliary Surgery Hospital, The Second Military Medical University, Shanghai 200438, China

**Keywords:** SPRY4-IT1, lncRNA, GBC, metastasis, EMT

## Abstract

Gallbladder carcinoma (GBC) is the most common malignancy of the bile duct and patients with GBC have extremely poor prognoses. Long non-coding RNAs (lncRNAs) are found to be dysregulated in a variety of cancers, including GBC. SPRY4-IT1 has been recently revealed as oncogenic regulator in many cancers. However, whether SPRY4-IT1 is involved in GBC progression remains largely unknown. To investigate the role of SPRY4-IT1 in GBC, we evaluated the expression SPRY4-IT1 in GBC tissues and cell lines, and investigated the effect of SPRY4-IT1 knockdown on cell proliferation, migration and invasion of GBC *in vitro*. Our result showed that SPRY4-IT1 was upregulated in GBC tissues. Further experiments revealed that SPRY4-IT1 knockdown significantly inhibited GBC cell proliferation. Furthermore, inhibitory effects of SPRY4-IT1 on cell migration and invasion were partly associated with EMT process. In conclusion, these data suggest that SPRY4-IT1 could be an oncogene for GBC, and may be served as a candidate target for new therapies in human GBC.

## INTRODUCTION

Gallbladder carcinoma (GBC) is the most common cancer of the biliary tract and the sixth most common gastrointestinal cancer [1–2]. Curative resection is the only cure for this highly lethal malignancy [3–4]. However, owing to its non-specific symptoms and highly invasive nature, most patients are at an advanced stage when they are diagnosed. A significant number of these patients ultimately die from metastatic disease [5]. At the molecular level, GBC arises from a series of genetic and epigenetic alterations that inactivate tumor suppressor genes and activate oncogenes [6]. However, the basic mechanisms underlying GBC initiation and progression remain largely unknown. Therefore, it is urgent to identify molecular actors that play a relevant role in GBC biology and may serve as targets for novel biological therapies.

Recently, long non-coding RNAs (lncRNAs) with length greater than 200 nucleotides have gained prominence [7]. lncRNAs were initially recognized to represent random transcriptional noise, have been implicated in numerous biological behaviors, such as epigenetic regulation, chromatin modification, transcription and post-transcriptional processing [8]. Increasing evidence has revealed the contribution of lncRNAs as proto-oncogenes, tumor suppressor genes and drivers of metastatic transformation [9–10]. The lncRNA SPRY4-IT1 is derived from the intronic region of the SPRY4 gene [11]. Data has indicated that SPRY4-IT1 was involved in cancer development through regulation of alternative splicing of its target genes or gene expression. SPRY4-IT1 expression has been found to be up-regulated in many solid tumors and has a tumor-promoting function [12–15]. However, the role of SPRY4-IT1 in GBC and the detailed molecular mechanisms remain poorly understood. In this study, we measured SPRY4-IT1 expression levels in GBC tissues and cancer cell lines and investigated the biological role of SPRY4-IT1 in GBC pathogenesis.

## RESULTS

### Increased expression of SPRY4-IT1 in GBC tissues and cell lines

**Figure 1:**
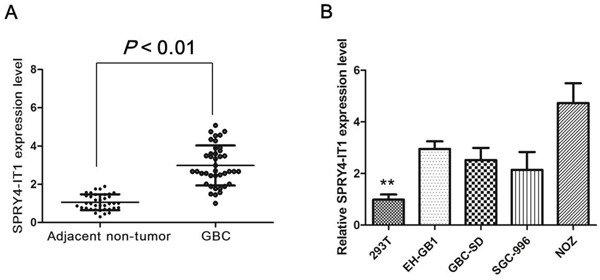
Expression of SPRY4-IT1 were increased in GBC tissues and cell lines **A.** Relative SPRY4-IT1 levels in GBC tissues and adjacent non-tumor tissues. **B.** Relative SPRY4-IT1 levels in GBC cell lines and 293T cell, *P<0.05; **P<0.01.

We firstly examined SPRY4-IT1 expression level in 38 paired GBC samples and adjacent normal tissues using qRT-PCR approach. As shown in Figure 1A, the SPRY4-IT1 level was significantly up-regulated in GBC tissue compared with corresponding adjacent non-tumor tissues (*P*<0.01), indicating that SPRY4-IT1 expression may be related to GBC pathogenesis. SPRY4-IT1 expression was not associated with gender and age, however, SPRY4-IT1 expression was significantly associated with tumor sizes and tumor status, lymph node metastasis.

To further explore the function of SPRY4-IT1 in GBC, the expression level of SPRY4-IT1 was examined using qRT-PCR in four GBC cell lines. The expression of SPRY4-IT1 was observed to be higher in all four GBC cell lines compared with the 293T cell line. As shown in Figure 1B, the result of qRT-PCR revealed that NoZ cells showed higher expression of SPRY4-IT1; however, GBC-SD cells showed lower expression of SPRY4-IT1. Thus, we used NoZ and GBC-SD cells as a model to investigate the biological consequences of SPRY4-IT1in regulating cancer cell proliferation and invasion.

**Figure 2:**
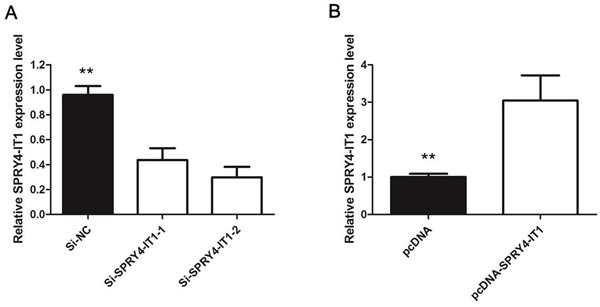
**A.** The qRT-PCR assay revealed that SPRY4-IT1 was efficiently downexpression by transfected with siRNA in NoZ cells; **B.** The qRT-PCR assay revealed that SPRY4-IT1 was efficiently overexpression in GBC-SD cells transfected with pCDNA-SPRY4-IT1.

To manipulate SPRY4-IT1 levels in NoZ cells, two separate SPRY4-IT1-specific siRNAs or a scrambled control were transiently transfected for loss of function analyses. Conversely, for gain of function studies, a pcDNA-SPRY4-IT1 vector was transiently transfected to ectopically overexpress SPRY4-IT1 in the GBC-SD cell line. Successful RNAi-mediated knockdown and ectopic expression of SPRY4-IT1 in NoZ and GBC-SD cells, respectively, were confirmed by qRT-PCR on harvested RNA 48 hours after transfection (Figure 2A and 2B).

### Knockdown of SPRY4-IT1 inhibited GBC cell proliferation and migration

**Figure 3:**
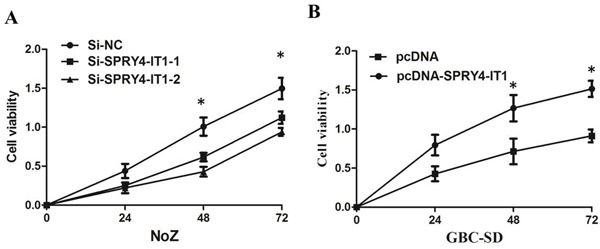
**A.** MTT assay showing knockdown of SPRY4-IT1 inhibited cell proliferation of NoZ cells compared to the negative control; **B.** MTT assay showingoverexpression of SPRY4-IT1 promoted cell proliferation of GBC-SD cells compared to the negative control.

**Figure 4:**
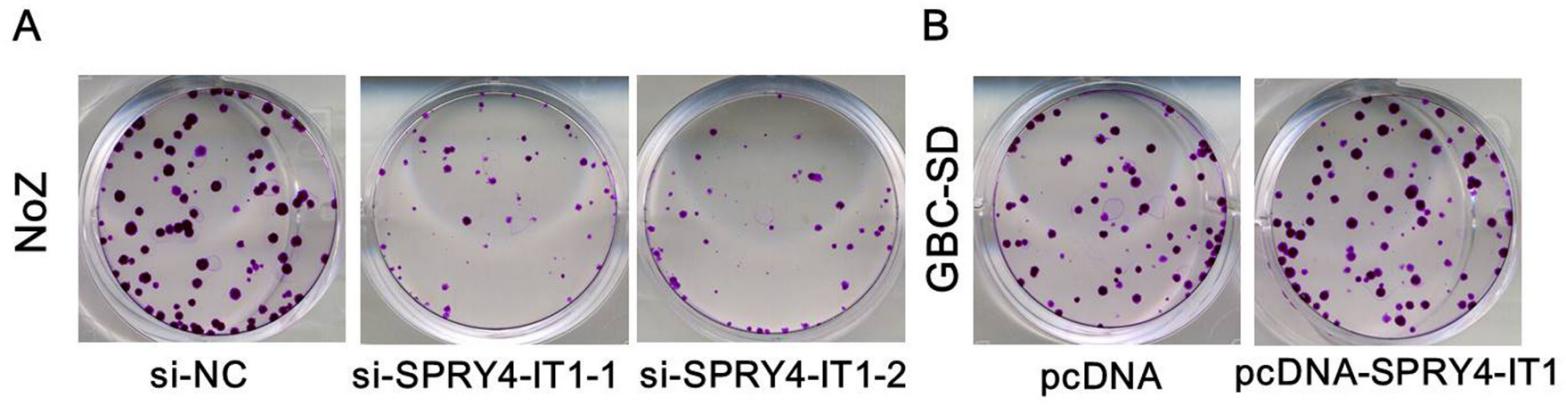
**A.** Colony-formation assays showed that silencing of SPRY4-IT1 significantly inhibited the colony-forming ability of NoZ cells compared to the negative control; **B.** Colony-formation assays showed that overexpression of SPRY4-IT1 significantly increased the colony-forming ability of GBC-SD cells compared to control.

To further examine whether SPRY4-IT1 is involved in GBC progression, *in vitro* functional analyses were performed. The results of MTT assay showed that knockdown of SPRY4-IT1 by siRNA significantly decreased proliferation of NoZ cells (*P*<0.05; Figure 3A). Furthermore, cell proliferation was also measured using a colony formation assay. Compared with the control cells, SPRY4-IT1 knockdown in NoZ cells resulted in markedly decreased colony formation abilities (*P*<0.05; Figure 4A). These findings indicate that SPRY4-IT1 may be closely associated with the proliferation of GBC cell lines.

**Figure 5:**
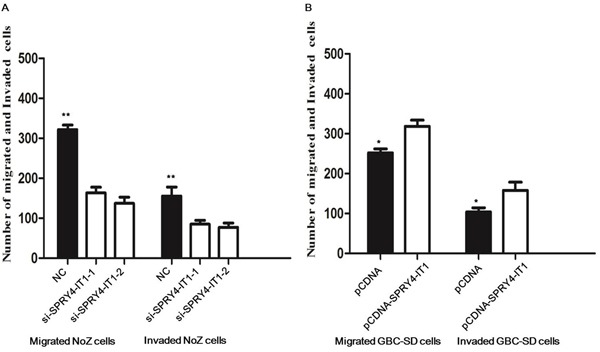
Effects of the SPRY4-IT1 on cell migration and invasion in GBC cell line **A.** Inhibition of migration and invasion of NoZ cells by SPRY4-IT1 siRNA; **B.** Upregulation of Migration and Invasion of GBC-SD cells by overexpression of SPRY4-IT1.

Because cell migration and invasion are essential prerequisites for cancer metastasis, we next employed transwell assay to evaluate the effect of differential SPRY4-IT1 expression on *in vitro* migration and invasion. Although si-NC-transfected NoZ cells showed robust *in vitro* migration, knocking down SPRY4-IT1 with either of the two siRNAs significantly inhibited *in vitro* migration and invasion (Figure 5A; *P*<0.01).

### Overexpressing of SPRY4-IT1 promoted GBC cell proliferation and migration

To assess the biological role of SPRY4-IT1 in GBC, we investigated the effects of SPRY4-IT1 overexpression on the proliferation of GBC-SD cells. MTT assays showed that the growth of GBC-SD cells transfected with pCDNA-SPRY4-IT1 was increased compared with control cells (*P*<0.05; Figure 3B). Colony formation assay results revealed that clonogenic survival was incresaed following overexpression of SPRY4-IT1 in GBC-SD cells (*P*<0.05; Figure 4B). The migration and invasion activity of SPRY4-IT1-overexpressing cells was significantly increased in GBC-SD cells (*P*<0.05; Figure 5B).

### SPRY4-IT1 influences GBC cell EMT

**Figure 6:**
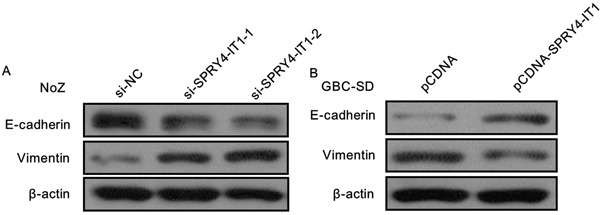
**A.** knockdown of SPRY4-IT1 expression downregulated E-cadherin expression and up-regulated Vimentin expression in NoZ cells; **B.** Overexpression of SPRY4-IT1 could increase E-cadherin and decrease Vimentin expression in GBC-SD cells.

As EMT process playing a key role in cancer cells invasion and metastasis, and our previous study indicated that lncRNAs also involved in cancer cells invasion via regulating EMT. In the present study, we determine the expression of the EMT-induced markers in SPRY4-IT1 overexpressed or downregulated GBC cells. The western blot results showed that overexpression of SPRY4-IT1 could increase E-cadherin and decrease Vimentin expression in GBC-SD cells, while knockdown of SPRY4-IT1 expression downregulated E-cadherin expression and up-regulated Vimentin expression in NoZ cells (Figure 6a and 6b), suggesting that SPRY4-IT1 contributes to GBC cells metastasis may partly via affecting EMT process, and further experiments are needed to elucidate the potential mechanism.

## DISCUSSION

Although previous studies have documented alterations of many oncogenes and tumor-suppressor genes involved in GBC, the molecular and genetic basis of GBC remain largely unknown. The prognosis of GBC is extremely poor, so it is urgent to understand the mechanisms underlying GBC progression. Although previous studies have characterized a few mammalian lincRNAs, which were reported to function in some important cellular processes, the functions of most annotated lincRNAs remain unexplored [16–17]. Meanwhile, altered lincRNA levels can result in the aberrant expression of gene products that may contribute to cancer biology. However, there are few studies on the functional roles of lincRNAs in GBC, and the overall pathophysiological contributions of lincRNAs to GBC remain unknown.

Increased SPRY4-IT1 expression has been reported in lung cancer, pancreatic cancer, and hepatocellular carcinoma [18–19]. In these tumors, SPRY4-IT1 may serve as a potential oncogene, and SPRY4-IT1 overexpression was associated with enhanced cell proliferation, reduced apoptosis, and increased cell migration [20]. However, the functions of SPRY4-IT1 in GBC were previously unknown. In this study, the qPCR analysis showed that SPRY4-IT1 was overexpressed in human GBC, compared with paired peritumoral tissues, which we believe represents a set of uncharacterized lincRNAs that may have important biological functions in this disease. Then we correlated SPRY4-IT1 levels with different clinicopathological factors of GBC tissues. We found that high SPRY4-IT1 expression was more frequently detected in GBC patients with larger tumor size, deeper invasion depth, positive lymph node metastasis, and advanced TNM stage. Additionally, SPRY4-IT1expression was markedly increased in GBC cell lines compared with 293T cells, suggesting that high SPRY4-IT1expression was closely associated with GBC carcinogenesis.

Our subsequent studies showed that knockdown of SPRY4-IT1 decreased cell proliferation and caused a dramatic decrease in colony formation in the NoZ cells. This result was confirmed by overexpression of SPRY4-IT1 in GBC-SD cell lines. MTT assays showed that the growth of GBC-SD cells transfected with pCDNA-SPRY4-IT1 was increased compared with control cells. Colony formation assay results revealed that clonogenic survival was incresaed following overexpression of SPRY4-IT1 in GBC-SD cells. Our results were consistent with the previous findings in other cancers. In lung cancer A549 cells, cell proliferation and colony formation were significantly inhibited in vitro after successfully depletion of SPRY4-IT1.

Metastasis is the main cause of mortality in cancer patients. The underlying mechanism for tumor metastasis and recurrence is very complex [21]. In addition, knockdown of SPRY4-IT1 inhibited the migration and invasion of GBC cells, which was consistent with the functional changes that occurred after silencing the expression of SPRY4-IT1 in GBC cells. Moreover, overexpression of SPRY4-IT1 significantly increased the ability of the migration and invasion of GBC-SD cells. EMT is a process of epithelial cells into mesenchymal cells trans-differentiation which is characterized by lost of cell-cell adhesion and acquired the traits of migratory and invasion. In the present study, we identified lncRNA SPRY4-IT1 as a novel player in modulating EMT progress. We found that knockdown of SPRY4-IT1 can suppress migratory and invasive phenotype of GBC cells by regulating EMT progress. Downregulation of SPRY4-IT1 in GBC cells remarkably increased the expression of the epithelial marker E-cadherin and meanwhile greatly decreased the expression of the mesenchymal marker vimentin, suggesting that SPRY4-IT1 might be a novel clinical marker for the prognosis of GBC and might represent a target for therapy.

Taken together, these researches indicated that SPRY4-IT1 is highly expressed in GBC. SPRY4-IT1 promotes the growth and migration of GBC cells, suggesting that SPRY4-IT1 may be an important contributor to GBC development.

## MATERIALS AND METHODS

### Patients and samples

Thirty-eight paired GBC tissue samples and neighboring noncancerous gallbladder tissues were obtained from patients who had underwent surgery at Eastern Hepatobiliary Surgery Hospital (Second Military Medical University, Shanghai, China) between 2010 and 2014, and were staged according to the tumor node metastasis (TNM) staging system (the 7th edition) of the American Joint Committee on Cancer (AJCC) staging system. Patients recruited to this study did not receive any pre-operative treatments. All specimens were immediately frozen in liquid nitrogen, and stored at −80 °C until RNA extraction. This study was approved by the Research Ethics Committee of Second Military Medical University, China. Informed consents were obtained from all patients.

### Cell culture

The human GBC cell lines EH-GB1, GBC-SD, SGC-996, and NOZ and the 293T cell line were purchased from the Institute of Biochemistry and Cell Biology of the Chinese Academy of Sciences (Shanghai, China). The cell lines were cultured in Dulbecco's modified Eagle's medium (Gibco BRL, Grand Island, NY, USA), supplemented with 10% fetal bovine serum (FBS, HyClone, Invitrogen, Camarillo, CA, USA), and 100 ug/ml penicillin and 100 μg/ml streptomycin (Invitrogen, Carlsbad, CA, USA). Cells were incubated at 37°C with 5% CO_2_.

### RNA extraction and reverse transcription

Total RNA was extracted from clinical samples and cell lines by TRIZOL reagent (Life Technologies, Foster City, CA, USA) and treated with DNase I (Invitrogen, Carlsbad, CA, USA) to eliminate potential DNA contamination. The GeneAmp RNA PCR kit (Life Technologies) was used to reverse-transcribe RNA to complementary DNA for the gene expression analysis.

### Quantitative reverse transcription polymerase chain reaction (qRT-PCR)

Real-time PCR analyses were performed with SYBR Premix ExTaq II kit (Takara, Dalian China). Results were normalized to the expression of GAPDH. The sequence of the primers were as following: SPRY4-IT1 (Forward: 5’-AGCCACATAAATTCAGCAGA-3’, Reverse: 5’-CGATGTAGTAGGATTCCTTTCA-3’) and GAPDH (Forward: 5’-GACTCATGACCACAGTCCATGC-3’, Reverse: 5’-AGAGGCAGGGATGATGTTCTG-3’). The qRT-PCR assays and data collection were performed on ABI 7500, and results were analyzed and expressed relative to threshold cycle values (ΔCt), then converted to fold changes using the 2−ΔΔCt method. GAPDH was used as an internal control.

### Plasmid generation

The SPRY4-IT1 sequence was synthesized and subcloned into the pCDNA3.1 vector (Invitrogen, Shanghai, China). Ectopic expression of SPRY4-IT1 was achieved through pCDNA-SPRY4-IT1 transfection, with an empty pCDNA vector used as a control. The expression levels of SPRY4-IT1 were detected by qRT-PCR.

### Cell transfection

Plasmid vectors (pCDNA3.1-SPRY4-IT1, and pCDNA3.1) for transfection were prepared using DNA Midiprep or Midiprep kits (Qiagen, Hilden, Germany), and transfected into cells. Two small interfering RNAs si-SPRY4-IT1-1 and si-SPRY4-IT1-1 were transfected into cells. Cells were grown in 6-well plates until confluent, then transfected with Lipofectamine 2000 (Invitrogen, Shanghai, China) according to the manufacturer's instructions. At 48 h post transfection, cells were harvested for qRT-PCR. The target sequences for the si-SPRY4-IT1 included: si-SPRY4-IT1-1 (CCCAGAATGTTGACAGCTGCCTCTT) and si-SPRY4-IT1-2 (GCTTTCTGATTCCAAGGCCTATTAA).

### Determination of cell proliferation and colony formation assay

Forty-eight hours after transfection, 3000 cells per well were seeded into 96-well plates. After 6, 24, 48, 72 and 96 h of culture, cell viability was measured using the Cell Proliferation Reagent Kit I (MTT; Roche Applied Science).

For the colony formation assay, a total of 500 cells were placed in a fresh six-well plate and maintained in media containing 10% FBS, replacing the medium every 4 days. After 14 days, cells were fixed with methanol and stained with 0.1% crystal violet (Sigma-Aldrich). Visible colonies were manually counted. For each treatment group wells were assessed in triplicate.

### Cell migration and invasion assays

For the transwell assays, at 48 h post-transfection, cells in serumfree media were placed into the upper chamber of an insert (8-μm pore size; Millipore, Billerica, MA, USA). Medium containing 10% FBS was added to the lower chamber. After incubation for 24 h, the cells remaining on the upper membrane were removed with cotton wool, whereas the cells that had migrated or invaded through the membrane were fixed with 100% methanol and stained with 0.1% crystal violet, imaged, and counted using an IX71 inverted microscope (Olympus, Tokyo, Japan). Experiments were independently repeated three times.

### Western blot

Total protein extracts from cells were separated on 10% SDS-PAGE gels. Antibodies against E-cadherin and Vimentin (1:1,000 dilution) was purchased from Cell Signaling Technology (CST, MA, USA). Anti-β-actin (1:5000, Sigma) was used as the internal control. Bands were visualized using Thermo Supersignal West Pico Chemiluminescent substrate according to the manufacturer's instructions. The intensity of bands was determined by using Quantity One software, and the quantitative analyses of gray-scale value of each target protein vs that of individual β -actin were performed.

### Statistical analysis

SPSS 17.0 software was used for statistical analysis. All values were shown as means ± SEM of at least three independent experiments. For the analysis of differences between groups, independent-samples t test or one-way ANOVA was performed, and differences were considered significant for P < 0.05.
